# Modern-Day Green Strategies for the Removal of Chromium from Wastewater

**DOI:** 10.3390/jox14040089

**Published:** 2024-11-03

**Authors:** Komal Pandey, Baljeet Singh Saharan, Ravinder Kumar, Dilfuza Jabborova, Joginder Singh Duhan

**Affiliations:** 1 Department of Microbiology, Chaudhary Charan Singh Haryana Agricultural University, Hisar 125 004, India; pandeykomal444@gmail.com; 2 Department of Microbiology, Kurukshetra University, Kurukshetra 136 119, India; 3 USDA-ARS Root Disease and Biological Control Research Unit, Washington State University, Pullman, WA 99164-6430, USA; 4 Helmholtz Centre for Environmental Research—UFZ, Department of Environmental Biotechnology, Permoserstrasse 15, D-04318 Leipzig, Germany; 5 Department of Biotechnology, Chaudhary Devi Lal University, Sirsa 125 055, India; rsulakh@gmail.com; 6 Institute of Genetics and Plant Experimental Biology, Uzbekistan Academy of Sciences, Qibray 111 208, Uzbekistan; dilfuzajabborova@yahoo.com

**Keywords:** chromium, phytoremediation, rhizoremediation, mycoremediation and bacterial remediation

## Abstract

Chromium is an essential element in various industrial processes, including stainless steel production, electroplating, metal finishing, leather tanning, photography, and textile manufacturing. However, it is also a well-documented contaminant of aquatic systems and agricultural land, posing significant economic and health challenges. The hexavalent form of chromium [Cr(VI)] is particularly toxic and carcinogenic, linked to severe health issues such as cancer, kidney disorders, liver failure, and environmental biomagnification. Due to the high risks associated with chromium contamination in potable water, researchers have focused on developing effective removal strategies. Among these strategies, biosorption has emerged as a promising, cost-effective, and energy-efficient method for eliminating toxic metals, especially chromium. This process utilizes agricultural waste, plants, algae, bacteria, fungi, and other biomass as adsorbents, demonstrating substantial potential for the remediation of heavy metals from contaminated environments at minimal cost. This review paper provides a comprehensive analysis of various strategies, materials, and mechanisms involved in the bioremediation of chromium, along with their commercial viability. It also highlights the advantages of biosorption over traditional chemical and physical methods, offering a thorough understanding of its applications and effectiveness.

## 1. Introduction

Heavy metals play a significant role in industrial development but cause environmental pollution. The pollution caused by heavy metals has seriously threatened living organisms in an ecosystem. Heavy metals are exceedingly poisonous and non-biodegradable and accumulate in the food chain in miniature amounts. Therefore, industries including electroplating, aerospace, and metal finishing are among the most hazardous in terms of the effluent addition of heavy metals. For many decades, metal toxicity has posed a significant environmental issue due to its propensity for bioaccumulation and lack of biodegradability, thereby representing a critical challenge for both environmental and public health [1,2,3]. The possible health risks that heavy metals pose to the environment are receiving more and more attention from researchers. The primary contributors to heavy metal pollution include mining activities and wastewater from metallurgical processes, prompting the need for effective and cost-efficient metal removal methods and resulting in the advancement of innovative separation technologies. The hunt for novel technologies for removing harmful metals from wastewater has focused on biosorption, based on the metal-binding capabilities of diverse biological materials [4,5].

Several metals, including magnesium (Mg), nickel (Ni), chromium (Cr), copper (Cu), calcium (Ca), manganese (Mn), and sodium (Na), as well as zinc (Zn), are essential for metabolic and redox functions. Chromium-contaminated wastewater released directly into the environment poses a significant threat to human health and has adverse ecological effects. Although chromium is generally not a significant atmospheric pollutant, it may enter the air through industrial emissions and the burning of fossil fuels. Once it becomes airborne, chromium can return to soils and water bodies through precipitation or settling of particulates. Atmospheric Cr is usually associated with particulate matter and is found in Cr (III) and Cr (VI) forms. Chromium can be a hugely hazardous water contaminant, depending on its oxidation state [6]. Cr (III) may change the structure and activity of enzymes by reacting with their carboxyl and thiol groups. Intracellular cationic Cr (III) complexes electrostatically interact with the negatively charged phosphate groups of DNAs, potentially affecting transcription, replication, and mutagenesis [7].

Cr (VI) is water-soluble across various pH levels and can permeate cell membranes in both prokaryotic and eukaryotic organisms, potentially leading to DNA mutations by promoting the accumulation of reactive oxygen species. These reasons warrant an urgent need to remove Cr from the wastewater. As a result, remediation approaches for Cr (VI) have been intensively researched to establish a cost-effective, efficient, and safe process that does not generate harmful waste [8,9]. The literature is enriched with a wide range of methods and techniques for Cr removal. Among them, biosorption is a highly recommended, quick, and straightforward approach for removing pollutants from water, with high efficiency and the potential for contamination recovery. The “bio” prefix denotes the presence of a biological organism (which includes bacteria, fungi, and algae), implying that biosorption is an ecologically safe treatment method. The term “biosorption”, which was first used in 1951, refers to the binding or removal of various organic chemicals from aqueous solutions, including metals, fertilizers, and organic solvents, synthetic colours, insecticides, and pesticides [10]. It safeguards metals, dyes, odour-causing compounds, and other organic and inorganic species by utilizing live or dead biomass or its derivatives. Biosorbent research has revealed that living and dead microbial cells can take in metal ions and provide a potentially less expensive alternative to conventional absorbents [11,12].

Biosorption technology has become crucial for removing metal ions and organic molecules. The chemical composition of microbial cells primarily influences the biosorbent activity of metallic ions. The biosorption mechanisms may include ion exchange, complexation, coordination, adsorption, electrostatic interaction, chelation, and microprecipitation, though each mechanism contributes to varying extents. Further research and development are necessary to create adaptable, reliable, and cost-effective biological solutions for water treatment. This review aims to elucidate the present landscape of biosorption research while juxtaposing results from diverse studies [13,14].

### 1.1. Bioremediation of Other Heavy Metals

Most metallic elements and some metalloids with more than 5 gcm^−3^ densities are classified as heavy metals. Among the heavy metals that are widely recognized are chromium (Cr), cobalt (Co), copper (Cu), cadmium (Cd), arsenic (As), gallium (Ga), germanium (Ge), iron (Fe), mercury (Hg), lead (Pb), nickel (Ni), thallium (Tl), selenium (Se), and manganese (Mn). The manufacturing industry consistently discharges heavy metals, including those discussed herein, into the environment, especially into aquatic ecosystems, at levels that exceed permissible limits set by regulatory bodies. All metals are remediated by different mechanisms, as shown in Table 1.

We mainly study chromium because it is one of the sixteen most hazardous heavy metals that negatively impact human health while being an essential element. One of the most important sources of environmental pollution is the hexavalent form, which is also well-known for its toxic, carcinogenic, and mutagenic effects on people and other living things. It has been shown to cause nephrotoxic malignant neoplastic illness.

### 1.2. The Presence and Chemistry of Chromium in Nature

**Figure 1:**
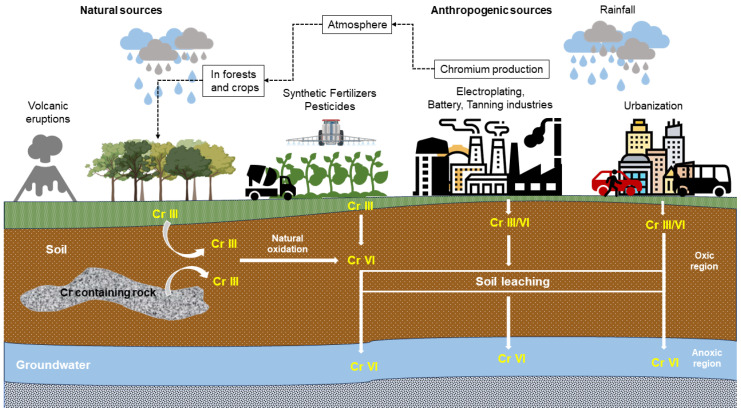
Various methods for releasing heavy metals (Cr) into soil and water systems.

Chromium has four major types, but generally, it exists in trivalent chromium Cr (III) or hexavalent chromium Cr (VI). Cr (III) is converted into more toxic Cr (VI) by oxidation under natural conditions or chlorination disinfection during drinking water treatment. Chromium is widely used in industrial production, such as in the electroplating, tanning, and dyeing industries, mining, coal production, batteries, pulp, and papers [23], as shown in Figure 1. Stable chromium exists in complex forms that incorporate organic pollutants, whereas Cr (III) is recognized as a vital trace element necessary for sustaining normal glucose tolerance and facilitating glucose metabolism. High concentrations of Cr (III) are still toxic to people, fish, and plants. Cr (VI) is generally harmful, bioaccumulates, is soluble in a wide range of pH levels, and is highly mobile in the environment. Many industries utilize chromium and discharge it in waste streams, where its removal is essential. Therefore, Cr (VI) discharge should be adequately regulated to prevent its discharge without treatments. Heavy metals in aquatic environments or wastewater are causing considerable concern. One of the less expensive strategies for removing heavy metals is biosorption [24,25,26].

The increasing use of chromium in industry and the discharge of chromium-contaminated wastewater and solid wastes has led to environmental pollution, which negatively affects ecosystems and is a primary global concern [27]. The tanning industry is considered a significant source of pollution, producing harmful gasses such as hydrogen sulphide. Basic chromium sulphate [Cr(H_2_O)_5_(OH)SO_4_] is the most common tanning agent used in tanning operations. Nonetheless, since only 60–70% of chromium is generally utilized for the treatment of hides and skins, there exists a significant concentration of unprocessed chromium salts in tannery effluent [28]. In electrolytic plating, Cr (VI) is frequently employed in welding, chromate painting, and other aerospace, automotive, and general engineering parts like gears and cylinders, which necessitate hard chromium coatings due to its superior solubility in water and its tendency to reduce to trivalent chromium when plated on metal surfaces spontaneously [9,29]. The nuclear industry has recently expanded the range of applications for hard chromium electroplating, for example, on the surface of cladding to reduce chemical interactions between nuclear fuel and steel cladding during reactor operations [30]. Numerous studies have investigated the removal of chromium from aqueous solutions by biosorption utilizing various adsorbents, with benefits including reduced sludge production, high efficiency, and the potential to use inexpensive and waste materials. Many genera like *Bacillus*, *Pseudomonas*, *Staphylococcus*, *Pediococcus*, and some species of *Klebsiella* have chromate reductase enzymes that help catalyze the reduction of Cr (VI) to Cr (III). Additionally, certain seaweeds, microalgae or marine algae, red algae, and brown algae are very effective biosorbents that may bind a variety of metals from aqueous effluents due to their low cost, availability in both fresh and saltwater, relatively high surface area, and high binding affinity. Seaweeds may absorb metals through chemical processes, including carboxyl, sulphonate, hydroxyl, and amino groups [31].

### 1.3. Effect of Chromium on Human Health and Environment

**Figure 2:**
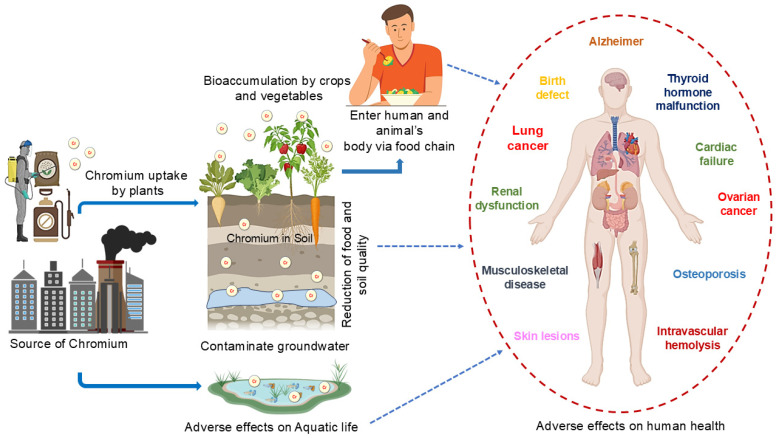
Chromium entry routes and the serious adverse effects of chromium on human health.

Chromium (VI) is a recognized waterborne contaminant due to its chemical characteristics, which enable its concentration level to rise in water. It becomes waterborne because of various human activities. During the propagation of the environmental nutrient cycle, it is taken in by many plants and animals. Cr-contaminated water exhibits toxicity even when measured in parts per billion (ppb) [32]. Traditional chromium tanning in tanneries produces large amounts of hexavalent chromium-containing effluent. Only 60–70% of the chromium required in the tanning process is utilized, and 30–40% ends up in wastewater. The release of chromium-laden effluent into the environment causes serious environmental and health issues. Therefore, it is critical to recover chromium from wastewater before it is discharged into the atmosphere [33,34]. There are many reasons to be concerned about the presence of textile dyes from industrial effluents in aquatic environments, since these contaminants reduce the ability of water bodies to self-purify by obstructing light, interfering with photosynthesis, and causing a decrease in oxygen concentrations [35]. There is no doubt that Cr (VI) molecules are both acutely and chronically harmful, even though Cr (III) is an essential component of the human body [36]. However, the presence of excess chromium can lead to a range of adverse effects, including headaches, light-headedness, eye and skin irritation, allergic reactions, neurotoxicity, dermal toxicity or allergies, immunotoxicity, ingestion-related issues, liver failure, kidney damage, nervous system impairment, or even collapsed lungs from oxygen deprivation; all these side effects are illustrated schematically in Figure 2 [37,38]. Human Rights Watch estimates that 90% of tannery workers die before reaching the age of 50, with many succumbing to cancer, most likely caused by exposure to harmful chemicals used in leather tanning [39]. When Cr concentration levels exceed the normative allowable threshold, this heavy metal becomes particularly detrimental to people, animals, and the environment [40,41,42]. In addition to contaminating water sources (including soil and groundwater), these pollutants also affect biotic elements like plants and animals [43,44].

### 1.4. Techniques to Remove Chromium from Wastewater

**Figure 3:**
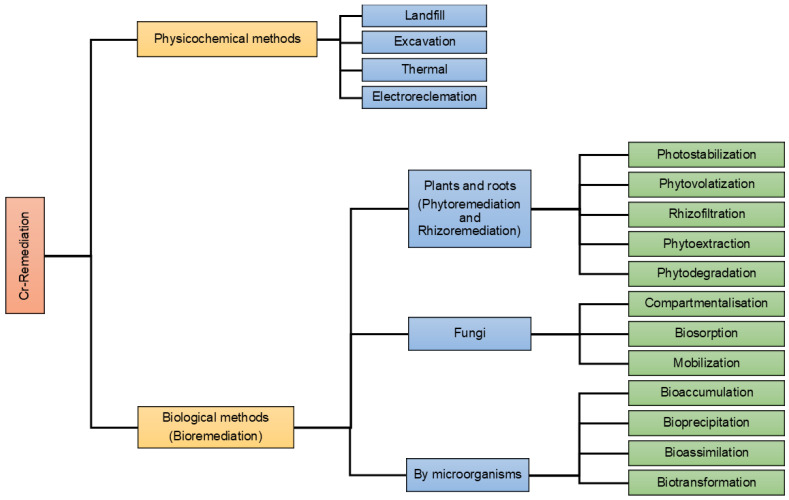
Methods available for the removal of heavy metals from wastewater. The methods can be broadly divided into physical, chemical, and biological methods based on the strategies used.

Various treatment technologies have been employed for the remediation of chromium-contaminated wastewater, including physicochemical methods (such as landfill, excavation, thermal, and electro-reclamation), chemical approaches (in situ chemical addition, electrokinetic), and biological methods (utilizing plants, root exudates, fungi, bacteria, algae, and their biomasses) [45,46,47,48,49] (Figure 3). Among electrochemical technologies, electrocoagulation has proven to be the most effective for removing pollutants and pathogens. This process involves the electrochemical generation of destabilizing agents from sacrificial anodes, such as aluminum and iron [50]. Membrane filtering systems were developed and implemented for the treatment of water and wastewater due to their superior removal efficiency, minimal pollution loads, and occasionally reduced energy consumption when compared to conventional methods [51]. Ion exchange technology has a broad range of applications for softening water and has integrated itself into new technical and industrial processes. The effectiveness of activated carbon and activated carbon composites as adsorbents to remove a variety of contaminants, including heavy metals and dyes, has been demonstrated by numerous studies [52]. Utilizing activated carbon for commercial wastewater treatment is not economically feasible. Carbonaceous adsorbents that have undergone surface modification are evaluated for hazardous ion (copper, zinc, chromium, and cyanide) adsorption in wastewater applications. Chromium metal ions were removed from the column at a 6.84 mg/g removal rate. The adequate removal capacity for Cr in the activated carbon column is two times higher than that of regular carbon and making activated carbon materials is costly and economically challenging. For that purpose, studies on heavy metal adsorption have been shifted towards abundantly available natural materials and some byproducts of industrial and agricultural processes [53].

Researchers have explored a variety of biosorbents, among which are rutins. Rutins, biosorbents of extracted polyphenols, are used in studies on the biosorption and desorption of Cr (VI) from solutions. Rutin and its resin reported maximal removal capacities of Cr (VI) of 26.3 mg/g and 41.6 mg/g, respectively. It may be possible to use rutin to create a natural biosorbent that is efficient at removing heavy metals from wastewater [54]. A cost investigation showed that biosorbents made from agricultural waste were less expensive than conventional adsorbents like activated carbon [55]. Agricultural wastes are a type of biomass widely distributed in nature and can effectively adsorb heavy metals on their surface. Studies have investigated using agricultural waste as a biosorbent, including rice husk and *Daucuscarota* L. waste biomass, palm flower, pomegranate husk, tamarind seeds, persimmon waste, *Pinus densiflora* leaves, *Leersia hexandra* Swartz biomass, *Eichhornia crassipes*, and *Aeglemar meloscorrea* shell [56]. Adsorption is the most efficient and adaptable method for heavy metal removal, even at deficient concentrations. However, the main barrier to industrial implementation is the high cost of adsorbents, often activated carbon [57]. Several biosorption studies over the past few decades have assessed the suitability of low-cost materials as biosorbents for synthetic dyes, which make up most wastewater produced by the textile industry. The ease of use, effectiveness, affordability, and straightforward separation technique inherent in membrane technologies enhance their appeal, particularly for use in wastewater treatment facilities in developing countries [58,59].

### 1.5. Drawbacks of Physical and Chemical Techniques to Remove Chromium

Although some physical and chemical treatment procedures are simple, fast, and may help metal recuperation, many still do not meet the demands of high operational costs, high energy consumption, and the production of secondary pollutants. The various pH solutions and the chemical makeup of the absorbents influence adsorption. It is effective for Cr (IV) and Cr (VI) but ineffective for Cr (III). The disadvantages of reverse osmosis include the need for high pressure, the likelihood of membrane degradation, and the high cost. Because its resins are so costly, ion exchange is sensitive to the presence of particulate contaminants. In electrochemical methods (oxidation-reduction), under adverse circumstances, the likelihood of a reverse reaction, i.e., the conversion of Cr (III) to Cr (VI), is very high. Chemical precipitation is undesirable because of their high solubility; chromium salts are difficult to separate from aqueous solutions; precipitation of soluble Cr (VI) is challenging in the presence of organics and frequently ineffective at low chromium concentrations [60,61].

While designing a sustainable and cost-effective process for metal removal, it is essential to consider all these associated drawbacks. However, biosorption presents a promising alternative to traditional clean-up methods. Low cost and minimal waste generation make microbial systems highly suitable for metal biosorption. As a result, biosorption is expected to provide an efficient, economical, and environmentally friendly solution for removing heavy metals from contaminated environments [55], which is discussed in detail here.

## 2. Bioremediation

As discussed above, physical and chemical remediation of heavy metals has many drawbacks. So, adopting more potent, economically and ecologically friendly bio-adsorbents that remove heavy metals without harming our environment and ecosystems is essential. Bioremediation includes rhizoremediation, bacterial remediation, mycoremediation (fungi), and phytoremediation (algae) [62,63,64,65,66]; they are all potentially used in bioremediation and discussed in detail as follows.

### 2.1. Rhizoremediation

Rapid urbanization, industrialization, organic pesticides and fertilizers, and associated pollutants like heavy metal run-off are the primary sources of agricultural land and groundwater contamination. It is an urgent environmental necessity to overcome this problem using a sustainable bioremediation process [67].

Roots are the first and foremost part of the soil that uptake and translocate heavy metal pollutants from the soil to the surrounding environment with the help of rhizosphere microorganisms, commonly known as plant growth-promoting rhizobacteria (PGPR) [68]. The soil rhizosphere microbes like Firmicutes, Actinomycetes, Proteobacteria and mainly PGPR like *Bacillus*, *Arthrobacter*, and *Pseudomonas* are promising microbes for sustainable agricultural practises that help remediate the heavy metal toxicity from the soil, and that process is known as rhizoremediation [69]. Rhizoremediation is a process in which the root structure is mainly affected. The root apex and the root’s younger parts actively absorb minerals, water, and inorganic substances, together with heavy metals, through the rhizodermis. Additionally, rhizoremediation is the process of a plant’s rhizosphere, or the small top region of the soil, interacting with soil microbes to create a situation in which both entities benefit (like mutualism)—the plant receives protection and aid in the fixation of nitrogen, the microbes receive the food they need in the form of root exudates. By supplying substrates, primarily carbon sources like glucose and fructose, which can trigger the metabolic pathways of the bacteria, the roots aid in boosting the microbial activity of the soil [70].

Under heavy metal stress, plants primarily modify their physiological properties, such as cell wall structure and the impregnation of secondary metabolites in the exodermis, endodermis, and rhizodermis, which are in direct contact with soil heavy metal pollutants. The formation of suberin lamellae and casparian bands in the exodermis and endodermis acts as an apoplastic barrier, protecting the roots by preventing the radial transport of heavy metals into the vascular bundles [71].

**Figure 4:**
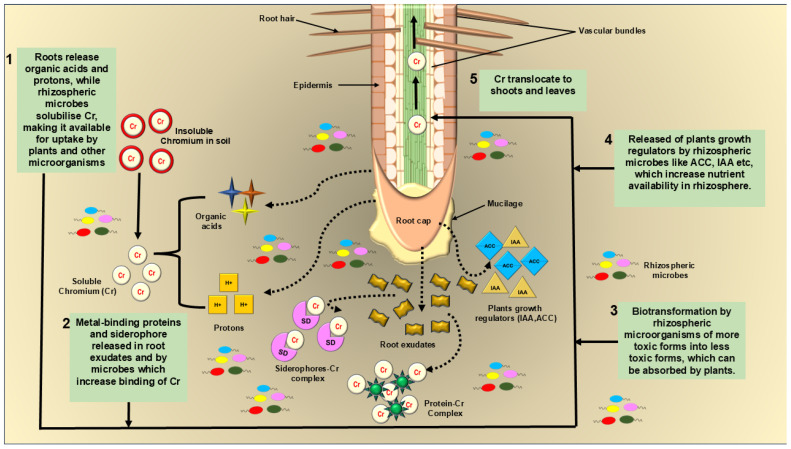
The diagram shows a rhizoremediation process in which rhizosphere microbes help plant growth and survival in Cr-containing soil and convert insoluble Cr into soluble Cr by producing components like organic acids and protons.

Recently, heavy-metal-tolerant plant-growth-promoting (HMT-PGR) microbial consortia have been used in several studies to evaluate the remediation effects of polluted sites. Still, there is a need for more exploration of the interaction between plants and microbes for bioremediation methods. HMT-PGR microbes in the rhizosphere region remove heavy metals from the soil and encourage plant growth by stimulating various components [72]. Elements like organic acid (gluconic, acetic, malic, and oxalic acids) convert insoluble heavy metals into soluble metals from polluted soil. Plant roots release root exudates, producing protons (H^+^) and enzymes, enhancing the heavy metal’s bioavailability. It also forms metal complexity by releasing organic components, which many soil microorganisms utilize as food and energy sources. Root exudates also release metal chelators like siderophores, metal-binding proteins that intracellularly bind heavy metals [73], briefly described in Figure 4. Soil micro-organisms also release extracellular polymeric substances (EPS) like lipopolysaccharides, glycoproteins, and polysaccharides, which have several anion functional groups that help to remediate heavy metals from soil.

Under heavy metal stress, microorganisms produce phytohormones like 1-aminocyclopropane-1-carboxylate (ACC) and indole acetic acid (IAA), which promote the formation of lateral and adventitious roots and enhance root elongation, thereby improving plant growth in such conditions [74]. Siderophores, metal chelators typically used for iron, can also bind to other metals such as Cr, Cd, Cu, and Pb, reducing their toxicity by lowering their concentration. Plant growth hormones like ACC deaminase and ethylene play crucial roles in physiological processes such as fruit ripening, blooming, and sprouting, contributing to improved plant resilience under metal stress [75,76]. The rhizoremediation process proceeds slowly in nature due to limiting variables, such as the variety of pollutants present in the soil, organic matter, bioavailability, energy sources, temperature, pH, and bacteria involved. In addition to the above factors, the benefit of rhizoremediation depends on the plant’s ability to withstand and degrade environmental pollutants. It is also essential to establish the maximum number of contaminants that can accumulate and be detoxified within the plant without endangering its health. It can also be used as a part of sustainable agricultural practises.

### 2.2. Phytoremediation

**Figure 5:**
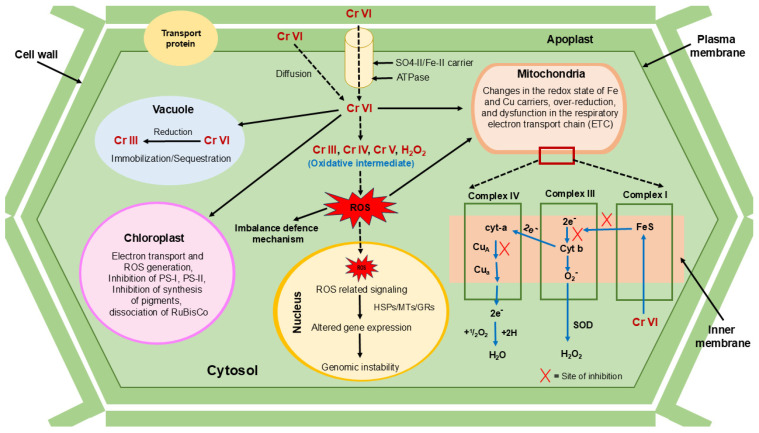
A hypothetical overview of the effects of chromium at the cellular level in plant cells.

Chromium majorly exists in four different oxidation forms: Cr (III), Cr (IV), Cr (V), and Cr (VI). In heavy metals, the Cr intermediate produces plant stress by producing ROS, which affects plants’ nucleus and mitochondria functions. Plants are majorly affected by Cr (VI) because its mobility toward plants is high compared to Cr (III), which is why Cr (VI) is also more toxic than Cr (III). Plants take Cr with the help of carriers like sulphates, which affect the plants’ defence mechanisms. Cr produces signs like electron transport chain (ETC) dysfunction in mitochondria, inhibition of PS-I and PS-II, pigment dissociation in RuBisCO in the chloroplast, and altered gene expression in the nucleus of plants, which are shown in Figure 5. Chromium is highly toxic to flora and, when considering food chains, poses risks to both animal and human health [77].

To overcome this heavy metal toxicity, plants use different mechanisms to remove toxicity, such as phytoremediation. Phytoremediation removes contaminants like organic pesticides or heavy metals from the soil, groundwater, and sediments with the help of plants and plant-associated microorganisms. It is a natural, efficient, cost-effective, and in situ process of bioremediation performed with the help of solar energy and soil micro-organisms [78].

**Figure 6:**
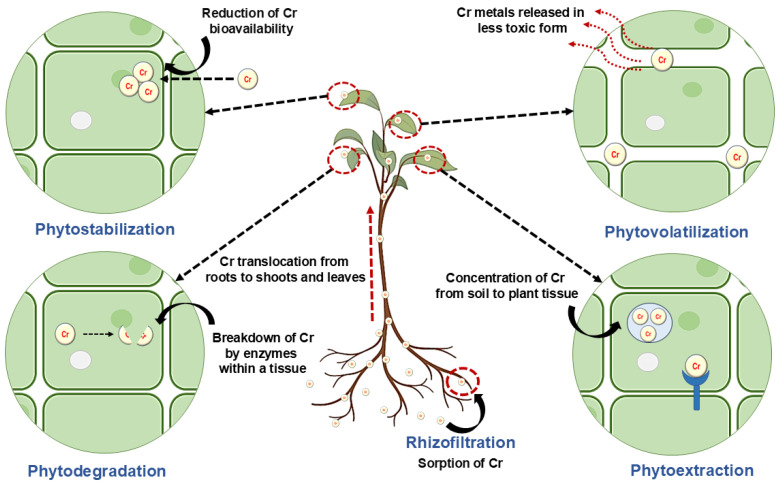
Different phytoremediation processes.

Phytoremediation includes mechanisms like hemofiltration, phytodegradation, phytoextraction, phytostabilization, and phyto-volatization, as shown in Figure 6. The first step of phytoremediation is phytoextraction, in which heavy metals are taken from contaminated water or soil by the plant root and translocated and accumulated into plant shoots. The second step is phytofiltration, in which rhizofiltration (plant root) and blasto-filtration (plant seed) absorb the heavy metals and minimize groundwater contamination. The next step is phytostabilization and phyto-immobilization, in which plants, with the help of roots, limit the availability of heavy metals in soil and groundwater, which prevents heavy metal migration in the food chain. Next is phytodegradation, in which heavy metals are degraded by the plant’s enzymes, like oxygenase and dehalogenase. Then, the final step, phytovolatilization, releases volatile forms of heavy metals into the atmosphere. However, it is not completely volatile in the atmosphere; instead, it transfers from one region (soil or water) to another area, like the atmosphere [79,80,81]. Table 2 describes various rhizospheric microbe-assisted phytoremediation studies along with their mode of action.

### 2.3. Microbial Remediation

Bacteria, fungi, algae, industrial wastes, agricultural wastes, and other polysaccharide materials serve as biosorbents for removing heavy metals and dyes. Various biomaterials have generally shown strong biosorption capacities for various metal ions. Microorganisms such as *Bacillus*, *Pseudomonas*, and *Streptomyces* are effective metal biosorbents [90,91]. Fungal biosorbents include significant species like *Aspergillus*, *Penicillium*, *Actinomycetes*, *Saccharomyces*, and *Rhizopus* [92,93,94]. Algae, such as Sargassum, microalgae, cyanobacteria, and brown and red algae, are also well-known for their metal biosorption properties [95,96,97]. Toxicant recovery is feasible with the right choice of eluant. Acidic or alkaline solutions have frequently been shown to be successful recovery methods for toxicants [98]. This approach can incorporate various biomass sources, including biological wastewater treatment plant sludge, by-products from the fermentation industry, seaweeds, bacteria, fungi, algae, and agricultural residues such as rice husk, rice bran, and wheat bran [99].

### 2.4. Advantages of Microbial Biosorption

Biosorption can remove heavy metals from wastewater based on the capacity of various biological components to bind metals. Recently, biosorption has become a viable and successful alternative for treating low-strength wastewater. Most biosorbents were made from waste biomass from industry and agriculture, and some were made using microbial methods [100,101]. The cost of biosorption is typically low compared to physical and chemical adsorption methods, and it is simple to use and store. However, biomass can be altered or processed to increase selectivity and allow multiple heavy metals to be taken at a time. No additional nutrient requirement is needed in the biosorption process. It is also capable of treating huge volumes of wastewater and is efficient in a wide range of conditions, including temperature, pH, salinity, and various kinds of contaminants [102,103,104]. Transport and other straightforward processing fees comprise most of the cost [105,106]. The pH significantly impacts the tendency of biomass to absorb a solution, and the technique can be used in various pH settings. However, the mechanism is unaffected by temperature since the biomass remains dormant [107]. Concerning adaptability, binding sites can support a wide range of ions. The biosorption uptake level was extremely high.

According to some reports, some biomasses can hold a toxicant load almost equal to their dry weight, and uptake is typically quick. Additionally, one of the best biosorbent matrices can be regenerated and has the potential for reuse over several cycles. With the right choice of eluant, toxicant recovery is achievable. Acidic or alkaline solutions have frequently been proven to be successful toxicant recovery methods. High toxicant affinity results from favourable circumstances [108,109,110,111,112]. There are some benefits of biosorption over traditional treatment methods, such as low cost, high efficiency for diluted concentration solutions, a small amount of chemical and biological sludge, no additional nutrients needed, and the potential for metal recovery and biosorbents to regenerate themselves. Biosorption has been shown to effectively remove various heavy metals from wastewater, such as Cr, Cd, Cr, Pb, Hg, and As [113,114].

### 2.5. Economic Viability, Scalability and Commercialization

An inexpensive treatment option using a “low tech” and environmentally friendly technique was made possible by the emergence of biosorption as an interdisciplinary field in science and technology. Many suggested procedures have been patented for commercial application, driven by the promising advantages of biosorption, which include low operational expenses, high efficacy, and a diminished volume of chemical and biological sludge that requires management. Although some commercial-scale units and pilot installations have been built, most biosorption systems remain laboratory-scale despite undeniable advancements over decades of study. To extract or sequester metals from aqueous solutions, biosorbents such as AlgaSORB^TM^, which is made from freshwater microalga *Chlorella vulgaris* immobilized on silica, B.V. Sorbex biosorbent, which is made from a variety of sources, including macroalgae, AMT-Bioclaim^TM^, which is made from a *Bacillus* sp. immobilized with polyethyleneimine and glutaraldehyde, Bio-Fix Biosorbent, which is made from a variety of sources including algae immobilized in porous polypropylene beads, and RAHCO Bio-Beads, which are made from a variety of sources including peat moss immobilized within an organic polymer, have been developed. On the other hand, these items have not proven profitable in the long run. Biosorption technology is still in its early stages of development, and its commercial success will rely on a deeper comprehension of this process guided by a practical justification for its possible uses and commercial development [115,116].

### 2.6. Microbial Biosorption

**Figure 7:**
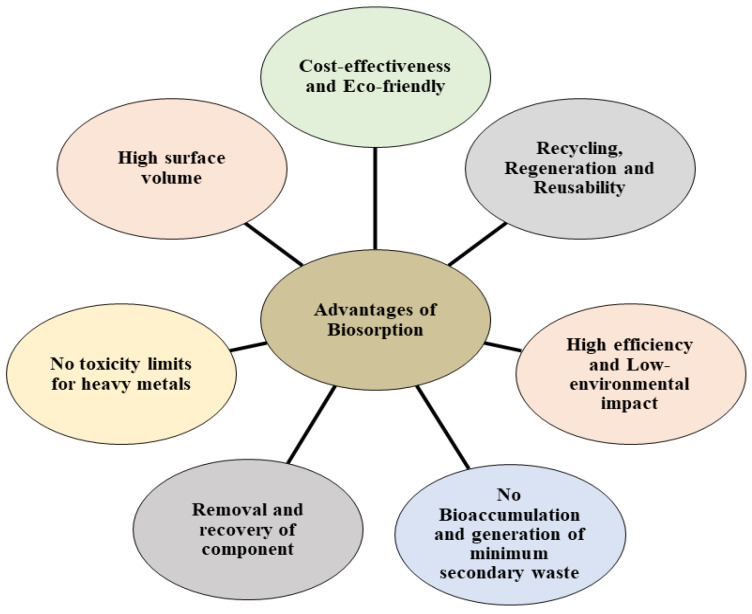
Advantages of biosorption and their secondary impact on the ecosystem.

Technologies for biological remediation are less expensive, safer, and more environmentally friendly, which encourages development, as shown in Figure 7. It has been discovered that removing environmental contaminants using biochar, microbial communities, industrial waste, and organic material alteration is an efficient method for improving soil fertility and quality. The primary disposal options that may be viewed as limitations within current biosorbent research encompass landfill, incineration, regeneration, and reuse, as well as safe disposal methods such as fertilizer application. After usage, the biosorbents must first be burned. In addition to significantly reducing the bulk and volume of waste biomass, incineration also recovers heavy metals and thermal energy, since the biosorbents are rich in biomolecules like cellulose and lignin [117,118].

Treating the waste biosorbents in landfills is the second step. Landfill procedures for biosorbents are straightforward and reasonably priced, much like residential trash. However, biosorbents containing dangerous metals can only be landfilled after desorption to prevent secondary contamination. After desorption, spent adsorbents can be either spread across land or buried underground, while the biosorbents themselves may eventually be entirely eliminated through natural degradation. For poor soil, the waste can be used as fertilizer. Since algae, microorganisms, forestry, and agricultural wastes are the raw materials used to make biosorbents, they include specific organic components or elements that improve the soil. In addition to helping with biosorbent disposal, using biosorbents as fertilizers can enhance soil quality. As a result, using leftover biosorbents as soil fertilizers offers a respectable way to get rid of them in the end [119,120].

The foundation for assessing the sorption of contaminants from water is the desorption and regeneration of utilized biosorbents. Desorption is a method of renewing biosorbents that are loaded with metal ions. The sorbent can be used again in further biosorption cycles by releasing the metal ions that were held on it into an aqueous solution [121].

#### 2.6.1. Types of Microbial Biosorption

Microbes are an emerging, cheap biotechnological tool that can be used for in situ bioremediation and other purposes. Biosorbents can be used in their viable or non-viable forms, and the entire system can be handled in batch, fed-batch, or continuous modes. Improvements in the technology of biosorption of Cr(VI) and other potentially harmful pollutants could be made possible by the isolation, selection, and genetic modification of novel microbes [122]. Biosorption refers to removing heavy metals by a passive binding process with living microorganisms such as bacteria, fungi, algae, and yeasts [123,124]. *Bacillus* sp., *Pseudomonas* sp., *Arthrobacter* sp., *Alcaligenes* sp., *Azotobacter* sp., *Rhodococcus* sp., and methanogens are among the genera of bacteria that aid in the elimination of heavy metals. Since the late 19th century, researchers have explored heavy metal absorption in aquatic species, an active process involving metabolic activity within living organisms [125].

##### Biosorption of Chromium by Bacteria

Microorganisms play a crucial role in the biological remediation of contaminated soils, waters, and industrial effluents. Low-cost microbiological methods for wastewater treatment of large quantities of complicated effluents containing Cr (VI) include bacteria, fungi, yeasts, microalgae, and cyanobacteria. The identification and exploration of new microorganisms, coupled with advancements in modern biotechnology and molecular techniques, enhance the properties of microorganisms—such as their adaptability and diversity—thereby enabling the biosorption of Cr (VI) as an alternative to traditional pollutant extraction methods. It is possible to use aerobic or anaerobic micro-organisms, live or dead, in the removal of Cr (VI) by varying the cell composition, shape, and mode of growth [126,127].

The antimicrobial metabolites produced by *Candida maltosa* and *Byssochlamys* sp. exert a microbiostatic impact. *Bacillus licheniformis*, *Bacillus megaterium*, and *Candida maltosa* eliminated 43.27, 75.48, and 92.75 mg/L of hexavalent chromium in 96 h, respectively [128]. *Byssochlamys* sp., on the other hand, eliminated a 50 mg/L concentration of hexavalent chromium in just 72 h. *Bacillus licheniformis*, *Bacillus megaterium*, *Byssochlamy* spp., *Candida maltosa*, and consortium haloalkaliphilic bacterial strains eliminated 41.20, 50.88, 64.44, 31.88, and 57.30% in 96 h, respectively, at 100 mg/L of hexavalent chromium. The best choice for bioremediation of chromium-contaminated leather industry wastewater and metal recovery in mining may be the *Byssochlamys* sp. [128]. Table 3 summarizes several studies on Cr heavy metal biosorption by bacterial species.

#### 2.6.2. Mechanisms of Bacterial Biosorption

**Figure 8:**
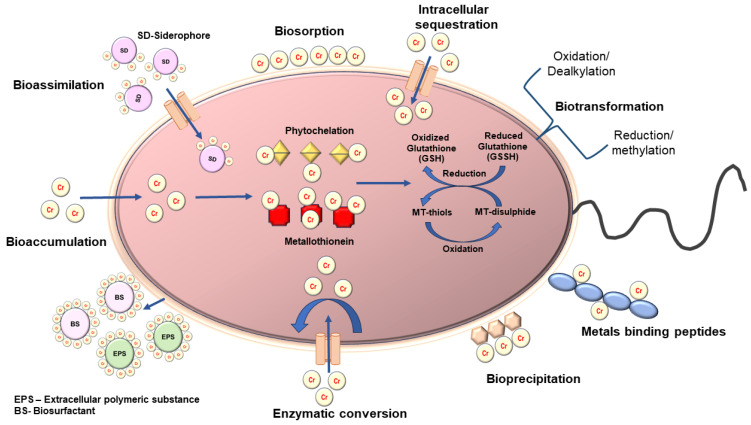
Schematic diagram showing the different mechanisms of bacterial remediation.

Chromium (VI) undergoes extracellular bioreduction at reaction sites located in the extracellular space, cytoplasm, and bacterial cell envelope using different methods like diffusion, complexation, surface adsorption, precipitation, and intracellular accumulation, as shown in Figure 8. Metal speciation and transportation may vary depending on their starting condition. For instance, metalloid dispersion and movement rely on the oxidation state and the ionic form; metals are often insoluble in their oxidation state [141,142]. Multiple strains of bacteria that predominate in *Bacillus*, *Pseudomonas*, and *Streptomyces* were described in heavy metal biosorption. The temperature, ionic strength, concentration, type of sorbate and biosorption, solubility or immobilization of biomass, and the existence of additional anions and cations in the growth medium are only a few examples of the variables [143,144]. Metal ions adhere to cell surfaces through electrostatic interactions with charged functional groups, a process that operates independently of metabolism [145]. Processes such as precipitation and surface complexation, ion exchange, or physical adsorption play the dominant role [146].

Active adsorption is the metabolism-dependent intracellular buildup of toxicants in living cells within the cytoplasm. When bound with heavy metals, these intracellular proteins can also lower the free ion concentrations within the cytoplasm, where detoxification occurs [147,148]. Heavy metals were transformed into a non-bioavailable state by connecting with metallothioneins (MTs), low-molecular-mass cysteine-rich proteins, and metallo-chaperones, as discussed below.

##### Metal-Binding Cysteine-Rich Peptides

The production of peptides rich in cysteine residues, metallothioneins (MTs), glutathione (GSH), or phytochelatin (PCs), is increased when cells are exposed to heavy metals at hazardous amounts. These are low molecular weight, non-enzymatic compounds resistant to acid precipitation and thermo-coagulation. These peptides’ primary function is to combine with divalent metals and metal-thiols to create complexes that include vital metabolites to counteract reactive oxygen species (ROS).

##### Metallothioneins (MTs)

All living things include metallothioneins (MTs), a class of well-preserved protein structures that function as antioxidants. They are high in cysteine residues and have a low molecular weight. The chemical structure of cysteines contains thiol groups (SH), which facilitate the binding of metal ions, including Cd^2+^, Fe^2+^, Hg^2+^, Cu^2+^, and Zn^2+^. The N-terminal region’s β domain and the C-terminal region’s α domain comprise the two distinct domains that make up MTs. Seven ions are bound per molecule, because the α domain contains eleven cysteine residues and connects four ions, whereas the β domain has nine cysteine residues connecting three divalent ions. Metallothioneins (MTs) fulfil numerous functions, including detoxifying heavy metals and providing a defence against reactive oxygen species (ROS). Therefore, MTs oversee both preserving the homeostatic cellular redox equilibrium and lessening the impact of oxidative stress brought on by these ions. Based on these properties, only metals can trigger the creation of proteins.

##### Glutathione (GSH)

All living things contain glutathione (GSH), also known as L-glutamyl-L-cysteinyl-glycine. Glutathione is a soluble antioxidant that is thought to be the most significant non-protein thiol. Its biochemical characteristics are attributed to the cysteine thiol group in its active site, composed of the three amino acids: glutamic acid (Glu), cysteine (Cys), and glycine (Gly). In addition to regulating its production, GSH is involved in several other activities, including the inactivation of reactive oxygen species (ROS), regulation of the intracellular redox status, transport of GSH-conjugated amino acids and other molecules, and storage of sulphur and cysteine. It is found in higher concentrations in the livers of animals. Its biosynthesis is comparable to that of protists, yeast, and plants. The nucleus and mitochondria each have a GSH reserve, which is essential or helpful in protecting these structures from ROS activity [149].

##### Phytochelatins (PCs)

Small peptides are rich in cysteine phytochelatins (PCs) and have the general structure (Glu-Cys) nGly (n = 2–11). PC synthase facilitates the processes involved in synthesizing PCs from glutathione (GSH). Through carboxyl and thiol residues, they allow ions to attach to different heavy metal ions. These PCs may be found in algae, cyanobacteria, nematodes, plants, and fungi. The PCs are more capable of attaching heavy metal ions (1 atom per cysteine basis) than MTs, even though they are categorized as MT-III. Because of multi-enzyme activities, the chemical bonds of type γ between Glu-Cys units offered a challenge to the groundbreaking work that focused on developing recombinant PCs in *E. coli*. The objective was to outperform non-recombinant microorganisms in adsorption procedures involving heavy metal ions by increasing the capability of these unique bacteria.

##### The “Surface-Cell Display” Protein

Since cell surface proteins are located at the interface of the cell and its surroundings, they represent an important class of biomolecules. Specific proteins can be bound to particular cell regions and anchored to the surface by cellular mechanisms. Both bacteria and *S. cerevisiae* employ a large number of systems. The manufacture of recombinant vaccines, antigens, antibodies, enzymes, and library peptides are among the biotechnology procedures that make extensive use of the potent approach known as “cell-surface display”, which involves expressing heterologous peptides on the cell surface [150,151].

Gram-positive bacteria like *Bacillus*, *Enterococcus*, *Streptococcus*, *Staphylococcus*, and many more have been shown to contain a high sorption or biosorption capacity, as they have thicker peptidoglycan layers. Also, the functional groups in the bacteria’s cell wall are responsible for the binding task, including carboxyl, phosphonate, amine, and hydroxyl. Therefore, the success of biosorption is also based on the composition of the bacteria’s cell wall [152,153].

Metal ions are taken up through a complex procedure of releasing exopolysaccharides (EPS), such as proteins, DNA, RNA, and polysaccharides, from the cell wall’s slippery layer. These are essential in preventing metals from penetrating the intracellular milieu, where ion exchange can occur. Several bacterial strains, including *Stenotrophomonas maltophilia*, *Azotobacter chroococcum*, and *Bacillus cereus* KMS3-1, were studied for commercial EPS production [154,155].

##### Biosorption of Chromium by Algae

Wastewater is a rich supply of vital nutrients such as nitrogen, phosphorous, carbon, and sulphur, which algae can use to grow. From unicellular to multicellular forms, algae are a vast and varied collection of essential plant-like organisms in aquatic and terrestrial environments. Algae can occasionally be discovered on snow and exposed rocks in conjunction with a fungus-like lichen. Applications of algae include fertilizer, energy production, pollution reduction, stabilizing agents, and food. In wastewater treatment facilities, algae are utilized in pollution control to minimize dangerous organic and inorganic impurities [156]. Directly discharging wastewater into the environment may pose severe dangers to health and the environment, so it must be carefully treated. Heavy metals and antibiotics have also been found to be removed from wastewater by algae via bioaccumulation, biodegradation, and biosorption. Microalgal treatment can supplement traditional procedures by producing value-added products [157].

Microalgal biomass has been used to remove heavy metals from wastewater or contaminated aqueous solutions. Heavy metal removal from wastewater can be performed by various methods, such as precipitation on the cell surface, adsorption by the cell surface, enzymatic reduction, chelation with specific proteins, and bioaccumulation in organelles like cell vacuoles [158]. Several research teams have studied the biosorption of heavy metals from water-based solutions using a variety of algae species. Investigations were made into a microalgal isolate called *Chlorella miniata*’s capabilities and method for removing Cr (VI). Quantitative analyses showed that eliminating Cr (VI) required biosorption and bioreduction. Desorption investigations revealed that Cr (III) filled most of the adsorption sites on the biomass, after which the adsorbed Cr (VI) was converted to Cr (III). The starting pH, biomass, and Cr (VI) concentrations were all critical determinants of the equilibrium time for Cr (VI)removal [159]. The first and most crucial feature of microalgae is their rapid development rate (fewer than ten days), which indicates flexibility and strain stability.

Furthermore, microalgae have a high surface-to-volume ratio, allowing them to expand more in less volume. Using microalgae in wastewater restoration saves energy by eliminating the mechanical/electrical power required for traditional aeration methods [160]. Several types of microalgae have been employed to remove heavy metals from heavy metal-contaminated solutions, and the results of biosorption studies using different algae are given in Table 4.

##### Biosorption of Chromium by Fungi

Fungal creatures are eukaryotic, and most fungi develop as hyphae, which are tubular filaments. A mycelium is an intertwined cluster of hyphae. The N-acetylglucosamine polymer chitin provides strength to the hyphae. Since they comprise charged groups, the walls and envelopes of bacteria and fungi have characteristics that make them ideal for biosorption. The use of yeast in biosorption procedures, specifically for Cr (VI), has not received much research. They are adaptable microbes, primarily the *Saccharomyces* species, because they can grow in aerobic and anaerobic settings. They can be used by both living and dead organisms because they are harmless microorganisms [167,168].

**Figure 9:**
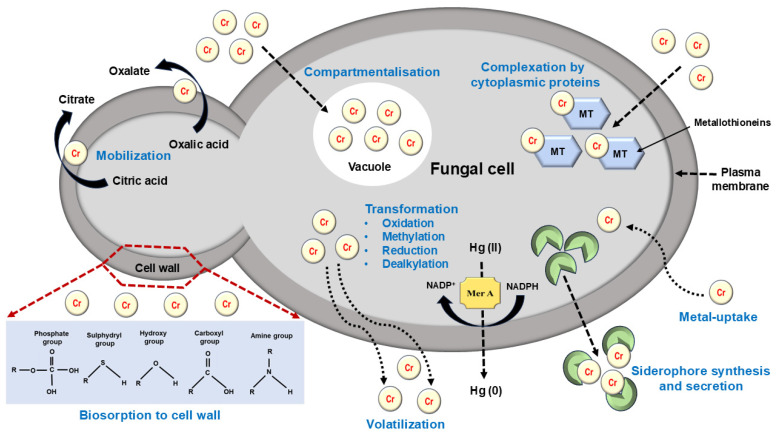
Different mycoremediation methods to remove chromium toxicity inside the fungal cell.

There are many different types of habitats for microbes. Among the numerous microorganisms with high opportunism, great adaptability, and rapid response to stressful situations, natural disasters, and harsh weather conditions are fungi. For bioremediation applications, organisms that can digest heavy metals and reduce environmental contamination are preferred. Fungi have both extracellular and intracellular biochemical and molecular mechanisms that depend on two processes: first, cellular uptake and compartmentalization; second, surface binding and complexation with functional groups, which is known as biosorption, and the binding of the metal to the cell surface via an ion exchange reaction as shown in Figure 9. Microorganisms exposed to heavy metals (HMs) have the potential to produce and secrete chelating compounds that have a metal-binding affinity. Metals from the extracellular milieu may also be precipitated by fungi-derived metabolites such as siderophores and organic acids, which will inactivate the metals. The model yeast *S. cerevisiae* may use hydrogen sulphide to create insoluble metal sulphides as an extracellular chelator. Cytoplasmic chemicals can render metals less or non-toxic, then complex and compartmentalize them inside the vacuole. This process is known as intracellular immobilization of metals. Glutathione, phytochelatins (PCs), and metallothioneins are the three main types of intracellular peptide-chelating metal ions (MTs). Since they are thiol compounds, they are the main players in MT participation in fungal HM detoxification and cellular resistance to HMs [169].

Microorganisms’ cell walls have evolved to bind materials like heavy metals (HMs) and shield the cells. Microorganisms have negatively charged cell surfaces due to the presence of different anionic substances like chitin in fungi and glucan in oomycetes. Metal biosorption is a group of chemical processes that can lead to metal binding to the cell wall. This kind of activity, which mostly employs carboxyl and phosphoryl groups to block the absorption of metal ions into cells, may also be attributed to biomolecules that include amine, hydroxyl, and sulphhydryl groups. Chemical processes, including methylation, dealkylation, oxidation, and reduction, are involved in other forms of intracellular metal inactivation. Ag and Cu reduction are among these reactions for fungal microbes. It has been discovered that fungal endophytes, such *as Curvulari ageniculata* P1 and *Lindgomycetaceae* P87, may decrease the mercury ion Hg (II). This reaction produces volatile forms of Hg, which allow the mercury to evaporate. A further instance of transforming hazardous metals into reasonably safe substances is the reduction of Cr (VI) by *A. niger*. There are two processes involved in this process: (i) Cr (VI) is adsorbed onto the cell wall by carboxyl, hydroxide, amine, amide, cyano, and phosphate groups, and (ii) Cr (VI) is reduced to Cr (III), which is water-soluble and less hazardous to cells. Enzymatic detoxification of metal ions involves oxidizing, reducing, methylating, and demethylating metal ions to make them less hazardous. An essential enzyme for Hg reduction, which makes it easier to convert Hg^2+^ to Hg^0^, is encoded by the merA gene. In mercury, Hg^2+^ is converted to volatile Hg0 via the merA gene and the enzyme mercuric ion reductase [170,171].

Filamentous fungi can extract concentrated heavy metal ions from liquid substrates, favouring them over other organisms for bioremediation. *Trichoderma autroviride*, *T. harzianum*, *T. virens*, and *Aspergillus niger* are only a few of the identified fungal species that are employed to clean up polluted environments. The ability of organisms to endure metal toxicity through a mechanism that directly interacts with specific metal species is defined as heavy metal resistance, as exhibited by fungi. Fungi biomass has a higher percentage of cell wall components than other biosorption agents, and these cell wall components have excellent metal binding characteristics, allowing them to absorb significant amounts of heavy metals even without physiological activity [172,173].

*Aspergillus niger*, *Aspergillus sydoni*, and *Penicillium janthinellum* were used as dead fungal biomass in the batch mode to study the biosorption of Cr (VI) ions from aqueous solution and electroplating effluent. *A. niger* removed Cr (VI) at a biosorbent dose of 0.6 g/50 mL, while *A. sydoni* and *P. janthinellum* removed it at a dose of 0.8 g/50 mL. However, the absorption capacity (mg/g) of Cr(VI) ions declined with the higher biosorbent dose [174]. *Penicillium griseofulvum* MSR1, *Rhodobacter sphaeroides* SC01, and *Pleurotus ostreatus* fungal biomass were used to remove chromium 79.9, 91.3, and 100% in 0.62, 96, and 360 h, respectively [175]. Chromium accumulation in plants causes various stresses and changes in peroxidase, ascorbate peroxidase, and acid invertase activity in pea plants [176,177]. Fungi are used in many industrial fermentation processes to remove metal ions from polluted areas [178,179,180]. Table 5 describes the studies on heavy metal biosorption by fungal species.

## 3. Conclusions and Future Perspectives

Microbial genetic engineering has been developed to enhance the ability of bacteria to cope with heavy metals. Extremophiles are particularly interesting to researchers due to their genetic and metabolic potential, which can be harnessed as micro-factories for removing heavy metals and other pollutants. Additionally, immobilizing bacterial biomass on suitable carriers may enhance porosity and physical and chemical stability. However, biosorption using immobilized microbial biomass has limitations, as the specific mechanisms of the process remain unclear. It is also important to acknowledge that waste microbial biomass generated from these sectors poses challenges for disposal. These waste microbial biomasses were used to create biosorbents, improving waste and solving their disposal issues. A biosorbent can be deemed successful once it has been utilized multiple times to remediate contaminants polluted by metals and dyes. Even if the microbial biosorbent can be effectively recycled over several cycles, the material’s final disposal must be considered. The typical solution for the final material’s disposal is to burn it or dump it in a landfill. However, the landfill option has lost some appeal because of rising landfill tax rates and probable limitations brought on by groundwater contamination. In addition, there is still much opportunity for investigation. In the coming decades, bioremediation with microalgae could begin as an integrated process with the concurrent generation of value-added products. The biomass produced by algae, bacteria, fungi, and yeast can make various value-added goods. Biofuels such as biodiesel, bioethanol, biohydrogen, biomethane, biofertilizers, food supplements, bioactive chemicals, pigments, biopolymers, and renewable energy are examples of such products.

## Figures and Tables

**Table 1:** A comparative list of different heavy metals used in bioremediation.

Microorganism	Heavy Metals	Microbial/Resistance Mechanism	References
*Microbacterium* sp. OLJ1 and Mycelial fungus *Talaromyces amestolkiae*	Pb	Biosorption	[15]
*Desulfovibrio desulfuricans*	Zn	Biosorption	[16]
*Rhizopus stolonifer*	Pb	Bioaccumulation	[17]
*Oceanobacillus profundus*	Pb, Zn	Biosorption	[18]
*Enterobacter cloacae*	Cd	Biosorption	[19]
*Sarcodia suiae* and *Pseudomonas aeruginosa* AT-01s Train	As	Biosorption	[20]
*Micrococcus* sp.	Pb, Cr, Cd	Biosorption	[21]
*Enterobacter cloacae*	Hg (II)	Biosorption	[22]

**Table 2:** Studies of various rhizosphere microbe-assisted phytoremediation.

Plant	Heavy Metal	Cr Stress Concentration	Micro-Organisms	Mode of Action	References
*Zea mays*	Cr (VI)	800–3000 ppm	*Streptomyces* sp.	ACC deaminase, IAA, P-solubilization, GA, and zeatin production	[64]
*Triticum aestivum* var. FSD-08	K_2_CrO_4_	1500 mg/mL	*Staphylococcus simulans* UT8, *S. saprophyticus*, *S. haemolyticus* NY2, *Enterobacter cloacae* UT25, *Brevibacterium* sp. AKR2, *Exiguobacterium indicum* LM8	Production of ACC deaminase, IAA, soluble proteins, and HCN	[74]
*Vigna radiata* L.	K_2_Cr_2_O_7_	5 10, 15 mg/kg	PGPR	Stop translocation of Cr about 21% from root to shoot	[77]
*Vetiveria zizanioides*	Cr (VI)	100 µg/mL	*Bacillus cereus*	Production of siderophore, IAA, ACC and solubilized phosphates	[82]
*Lens culinaris*	K_2_Cr_2_O_7_ and K_2_CrO_4_	500 µg/mL	*B. cereus* (EIV) and *B. cereus* (3a)	Production of phytohormones and antioxidant enzymes	[83]
*Zea Mays*, *Beta vulgare*, *Colocasia esculenta*, *Ficus carica*, *Parthenium argentatum*	K_2_CrO_4_	7.5, 5.1 and 2.5 µmol/mL	*Fusarium proliferatum*, *Penicillium radicum*, *Aspergillus fumigatus*, and *Rhizopus* sp.	Reduced translocation of Cr to the edible part of plants and detoxified Cr up to 95%	[84]
Green chilli	K_2_Cr_2_O_7_	500 µM/200 mL	*Cellulosimicrobium cellulans*	Phosphate mineralization and IAA production, as well as microbes, decreased the toxicity of Cr through a reduction process.	[85]
*Cynodon dactylon* (L.) Pers. and *Festuca arundinacea* Schreb.	CaCl_2_	3100.6, 1717, 368.6, 291.5, and 7.3 mg/kg	*Pseudomonas aeruginosa*	Increased bioavailability of low molecular-weight organic-acids (LMWOAs)	[86]
Marigold (*Tagetes erecta*)	Cr(NO_3_)_3_·9H_2_O	0.24, 0.16, 0.12, 0.08, 0.04 and 0.001 mmol/L	-	Hyperaccumulation of Cr and recovery of plant health and soil fertility	[87]
*Gossypium hirsutum*	Cr (VI) enriched sludge	100–500 mg/mL	*Streptomyces tritici* D5	Hyperaccumulation of Cr from soil and enhance plant growth	[88]
*Diectomis fastigiate* and *Vernonia cinerea*	Untreated mine waste effluent of Orissa mining corporation	2371 and 5500 mg/kg	-	Chromium hyperaccumulators in In situ condition	[89]

**Table 3:** Studies on chromium heavy metal biosorption by bacterial species.

Bacteria Used	Isolation Site	Initial Conc. of Chromium	Removal or Biosorption of Chromium (%)	Time (h)	References
*Actinomycete* strain *Kitasatosporia* sp.	Tannery wastewater	2 mg/L	99.0%	2	[25]
*Bacillus* sp. CRB-1	Tannery activated sludge	50 mg/L	100.0%	24	[27]
*Bacillus cereus*	Electroplating wastewater	50 mg/L	99.7%	120	[29]
Alkaliphilic *Bacillus subtilis*	Cr (VI) synthetic solutions	50 mg/L	99.0%	160	[90]
Haloalkaliphilic bacterial strains	Textile wastewater	300 ppm	56 ± 0.5%	5	[97]
*Bacillus licheniformis*, *Bacillus megaterium*, *Byssochlamys* sp. and *Candida maltosa*	Tannery industry	100 mg/L	41.2, 50.8, 64.4, 31.8,8; 57.3%, respectively	96	[128]
*Sphaerotilus natans*	Dichromate solution	20 mg/L	97.0%	120	[96]
*Rhizopus arrhizus*	Industrial wastewaters	250 mg/g	78.8 mg/g	72	[99]
*Pennisetum purpureum, Typha domingensis, Cyprus latifolius*, and *Echinochloa pyramidalis*	Tannery wastewater	1000 mg/L	99.3%	6	[129]
*Polygonum hydropiperoides*, *Azospirillum brasiliense*	Tannery wastewater	0.1762 mg/L	99.0%	6	[130]
*Rhodobacter*, *Hyphomicrobium*, *Leucobacter*, *Sphaerotilus* and *Acetobacterium*	Cr (VI) synthetic aqueous solution	8 mg/L	98.0%	3300	[131]
Microbial consortium	Tannery wastewater	2084.9 mg/L	95.2%	7872	[132]
*Bacillus* sp. JDM-2-1 and *Staphylococcus capitis*	Industrial wastewaters	4800 mg/L	89.0%	96	[133]
*Procambarus clarkii*	Industrial wastewaters	100 mg/L		24	[134]
*Pannonibacter phragmitetus*	Tannery wastewater	2200 mg/L		140	[135]
*Bacillus aerius* S1 and *Brevibacterium iodinum* S2	Tannery effluent	2 mM	99.0%	144	[136]
*Bacillus methylotrophicus*	Tannery effluent	0.1 mM	91.0%	60	[137]
*R*. *sphaeroides* SC01	Chromate solution	500 mg/L	99.0%	36	[138]
*Bacillus* sp. M6	Cr (VI) synthetic solutions	200 mg/L	68.8%	24	[139]
*Oceanobacillus* *oncorhynchi* W4	Cr (VI) wastewater	200 mg/L	72.4%	72	[140]

**Table 4:** Studies on heavy metal biosorption by algal species.

Algae Used	Isolation Site	Initial Conc. of Chromium	Removal or Biosorption of Chromium	Time (h)	References
*Sargassum* sp.	K_2_Cr_2_O_7_ synthetic solution	1000 mg/L	92.89%	3.5	[32]
*P. yezoensis* (red algae) *L. japonica* (brown algae)	Cr (VI) synthetic aqueous solution	100 mg/L	36 mg/g	12	[56]
Cyanobacteria	Cr (VI) synthetic aqueous solution	63.8 mg/L	30%	240	[91]
*Ceramium virgatum* (red algae)	K_2_Cr_2_O_7_ synthetic solution	0.03 mol/L	26.5 mg/g	1.5	[95]
*Phormidium* sp., a thermophilic cyanobacterium	Wastewater of textile and leather	100 mg/L	22.8 mg/g	6.6	[161]
*Sargassum tenerrimum*	Leather tanning	500 mg/L	88%	5	[162]
Microalga *Nannochloris oculata*	Cr (VI) synthetic aqueous solution	1000 mg/L	37.7 mg/g	3	[163]
*Sargassum* sp.	Cr (VI) synthetic aqueous solution	1000 mg/L	1.123 mmol/g	25	[164]
*Palmaria palmate* and *Polysiphonia lanosa* (red algae)	Cr (VI) synthetic aqueous solution	19 mM	4.94 and 8.64 mmol/g	6	[165]
*Pelvetia canaliculata* (brown algae)	Cr (VI) synthetic aqueous solution	2000 mg/L	72.7%	16	[166]

**Table 5:** Studies on heavy metal biosorption by fungal species.

Fungi Used	Isolation Site	Initial Conc. of Chromium	% of Removal or Biosorption	Time (h)	References
*Trichoderma* sp. BSCR02	Industrial effluent	200 mg/L	-	0.08	[92]
*Pleurotus ostreatus* fungal biomass	synthetic aqueous solutions	150 mg/L	100%	360	[93]
*Pannonibacter phragmitetus* LSSE-09	alkaline wastewater.	1000 mg/L	9.47 mg/g	26	[94]
*Rhodobacter sphaeroides* SC01	Cr (VI)-contaminated wastewater	500 mg/L	91.3%	96	[126]
*Saccharomyces cerevisiae*	synthetic aqueous solutions	10–100 mg/L	32.6 mg/g	-	[167]
*Penicillium griseofulvum* MSR1	Tannery effluent	67.8 mg/L	79.9	0.625	[175]
*Wickeramomyces anomalus*	Industrial effluent	100 mg/L	60%	26.6	[162]
*Aspergillus niger*	Mining wastewater	1.445 mg/L	0.0574 mg/g	15	[163]
*Aspergillus tamarii*	Steel and textile industries, Sewage water	50 mg/L	58.60%	-	[164]

## Data Availability

The data supporting this study’s findings are available from the corresponding author upon reasonable request.
